# A Hot-Spring Water Improves Inflammatory Conditions in an Injury-Induced Atopic Dermatitis Mouse Model by Regulating Skin Barrier Function

**DOI:** 10.3390/biomedicines13112707

**Published:** 2025-11-04

**Authors:** Yoko Naito, Abdullah Md. Sheikh, Jubo Bhuiya, Fatema Binte Abdullah, Jerin Fahmida, Shatera Tabassum, Hiro Tamegai, Kenichi Iwasa, Shozo Yano, Atsushi Nagai

**Affiliations:** 1 Department of Laboratory Medicine, Faculty of Medicine, Shimane University, 89-1 Enya Cho, Izumo 693-8501, Japan; yokonaito1@gmail.com (Y.N.); abdullah@med.shimane-u.ac.jp (A.M.S.); syano@med.shimane-u.ac.jp (S.Y.); 2 Department of Neurology, Faculty of Medicine, Shimane University, 89-1 Enya Cho, Izumo 693-8501, Japan; jubobhuiyan78@gmail.com (J.B.); jerinfahmida895@gmail.com (J.F.); tabassum@med.shimane-u.ac.jp (S.T.); htamegai@med.shimane-u.ac.jp (H.T.); ken1wasa@med.shimane-u.ac.jp (K.I.); 3 Department of Nephrology, Faculty of Medicine, Shimane University, 89-1 Enya Cho, Izumo 693-8501, Japan; m239401@med.shimane-u.ac.jp; 4 The Center for Integrated Kidney Research and Advance (IKRA), Faculty of Medicine, Shimane University, 89-1 Enya Cho, Izumo 693-8501, Japan

**Keywords:** atopic dermatitis, hot spring water, transepithelial water loss (TEWL), filaggrin, CD8+ T cells, stratum spinosum

## Abstract

**Background**: Atopic dermatitis (AD) is a common inflammatory skin condition in which skin barrier function plays a crucial role. Hot spring water is known for its beneficial effects on skin health. This study investigates the impact of a hot spring water on AD pathology, focusing on skin barrier function. **Methods**: Using the tape-stripping method, we induced an AD mouse model, treated the mice with either hot-spring water or tap water, and assessed time-dependent changes in skin barrier function, histology, and AD-related proteins. **Results**: Transepithelial water loss (TEWL) was significantly increased after tape-stripping, which began to decrease from day 2 in both treatment groups. On day 3, water loss was significantly decreased in hot-spring-treated mice than tap water-treated mice. Histological analysis revealed thickening and vacuolization of the stratum spinosum from day 2, becoming more pronounced on day 3 in tap-water-treated mice. However, in hot-spring-treated mice, the stratum spinosum was significantly less thickened, and the stratum granulosum was better formed. Immunostaining showed that transient receptor potential vanilloid 4 (TRPV4) levels decreased at day 2 but returned to baseline by day 3, with no significant differences between groups. Filaggrin, a key skin barrier protein, was markedly low in tape-stripped areas at day 0, but increased progressively, with a higher level in the upper epidermis of hot-spring-treated mice compared to tap-water-treated counterparts. Additionally, hot spring water treatment significantly reduced CD8+ T cell numbers and IL-4 cytokine levels, mitigating inflammation. **Conclusions**: Threfore, hot spring water enhances skin barrier recovery and reduces inflammation in AD.

## 1. Introduction

Atopic dermatitis (AD) is a common chronic inflammatory condition characterized by intense itching, redness, dryness, and the formation of patches or plaques on the skin [1,2]. Pathologically, it initially manifests as inflammatory cell infiltration, followed by epidermal spongiosis and micro-vesicle formation [2,3]. As the lesion becomes chronic, parakeratosis, acanthosis, and hyperkeratosis occur [1,3]. The exact cause of AD is not fully understood, but is believed to result from a complex interplay of genetic, environmental, and immunological factors [4]. Environmental factors, including exposure to allergens, injuries, irritants and pollutants can trigger or exacerbate the condition [5]. Individuals with a family history of atopic diseases such as asthma or hay fever are more likely to develop AD, suggesting a genetic predisposition. Additionally, genetic variations in immune-related genes, including those regulating interleukin (IL)-4, IL-13, and thymic stromal lymphopoietin (TSLP), can contribute to dysregulated type 2 immune responses and drive chronic inflammation and AD [6,7,8]. Also from an immunological perspective, AD is characterized by an imbalance between type 2 helper T (Th2) and Th1 immune responses, with acute lesions dominated by Th2 cytokines (IL-4, IL-5, IL-13) and chronic lesions showing a mixed Th1/Th2 profile [7,9]. The convergence of these genetic, environmental, and immunological components ultimately contributes to the persistent inflammation and skin barrier dysfunction characteristic of AD.

At the molecular level, AD manifests dysfunction in the skin barrier, immune dysregulation, and inflammation. Mutations in genes, which encode proteins that maintain the skin barrier, such as filaggrin, have been linked to an increased risk of developing AD [8,10]. These mutations typically decrease barrier proteins expression and compromise skin barrier functions, causing reduced moisture retention and skin dryness. Reduced barrier function also compromises protection against irritants and increases skin permeability, allowing allergens to readily enter the epidermal layer. Consequently, an immune reaction, especially of the Th2 type, is induced, which plays a key role in the pathogenesis and symptoms of AD [11]. For example, elevated levels of inflammatory cytokines including IL-4, IL-13, and IL-31 contribute to skin inflammation, reduction of skin barrier protein expression including filaggrin, recruitment of immune cells to affected areas, and result in itching and other symptoms of AD [8].

Previous studies have shown that transient receptor potential vanilloid 4 (TRPV4), a calcium-permeable ion channel protein, plays an important role in Th2 immune response and pruritus in AD [7]. TRPV4 is highly expressed in keratinocytes and macrophages in the skin, where it is implicated both in itching mechanisms and skin barrier regulation. Its expression in keratinocytes is markedly upregulated by Th2 cytokines, particularly IL-4 and IL-13, which are increased in AD [7,8]. Activation of TRPV4 in keratinocytes by mechanical or chemical stimuli promotes calcium influx, triggering the release of inflammatory and pruritogenic mediators such as endothelin-1 (ET-1). ET-1 acts as potent etch-inducing molecules that further amplify TH2-driven inflammation, creating a self-perpetuating cycle of itching and barrier disruption [9,12]. In a murine model, keratinocyte-specific deletion of *TRPV4* significantly decreased histaminergic itch, underscoring its pivotal role in AD-associated pruritus. Beyond its role in pruritus, TRPV4 activity influences keratinocyte differentiation and skin barrier activity [13]. In contrast, TRPV4 expressed in macrophages appears to exert anti-inflammatory effects by suppressing the polarization of macrophages towards the proinflammatory M1 phenotype. Consequently, it has been demonstrated that reduced TRPV4 expression in dermal macrophages of AD patients is associated with enhanced inflammation and more severe disease manifestation [9]. Together, these findings highlight complex interplay of TRPV4 ion channel regulation and Th2 inflammatory response in the context of atopic dermatitis pathology [9,14,15].

As described earlier, Th2 immune response and skin barrier function interact closely with each other in AD. Filaggrin is an essential structural protein of the stratum corneum, maintaining barrier integrity and hydration [10,16]. Its deficiency leads to increased transepidermal water loss (TEWL) and dry, scaly skin [10]. In AD, filaggrin deficiency compromises the epidermal barrier. Barrier dysfunction can arise either from inherited filaggrin gene (*FLG*) mutations or as an acquired defect secondary to inflammation. Loss-of-function mutations in the *FLG* represent the strongest known genetic risk factor for AD, particularly in patients with severe forms of the disease [17,18]. A compromised barrier facilitates the entry of environmental allergens, microbes, and irritants into the epidermis. These external agents stimulate keratinocytes to release cytokines such as thymic stromal lymphopoietin (TSLP), IL-25, and IL-33. TSLP activates dendritic cells, which promote naïve T-cell differentiation toward the Th2 lineage [19,20]. Th2 cells and innate lymphoid cells then secrete IL-4 and IL-13, cytokines that orchestrate the allergic cascade by inducing B-cell class switching to produce allergen-specific IgE and by recruiting eosinophils and mast cells [21]. These effector cells release mediators responsible for the characteristic itching and inflammation of AD. On the other hand, IL-4 and IL-13 directly downregulate filaggrin expression in keratinocytes [22,23]. They activate the JAK/STAT signaling cascade through binding to the IL-4Rα/IL-13Rα1 receptor complex, leading to phosphorylation of STAT6 and STAT3 [24]. These transcription factors inhibit OVOL1, a key regulator of filaggrin expression, by preventing its cytoplasmic-to-nuclear translocation [25]. In addition, IL-4 and IL-13 enhance the periostin–IL-24 axis, which further suppresses filaggrin synthesis [26,27]. This suppression occurs regardless of *FLG* mutation status. Consequently, the Th2-mediated inhibition of filaggrin exacerbates barrier dysfunction, allowing greater allergen penetration and sustaining chronic inflammation. This positive feedback loop between Th2 cytokines and filaggrin deficiency represents a central mechanism underlying the persistence and severity of AD [28,29,30,31].

Because both the Th2 immune response and skin barrier integrity play pivotal roles in the pathogenesis of AD, therapeutic strategies targeting these mechanisms are central to effective disease management [32,33]. Among available treatments, topical corticosteroids remain the first-line therapy for acute flare-ups, providing rapid relief of inflammation and pruritus [34]. More recently, targeted approaches have been developed to modulate the Th2 response [35,36]. Biologic therapies such as dupilumab, a monoclonal antibody that inhibits IL-4 and IL-13 signaling by blocking the shared α-subunit of the IL-4 receptor, have shown significant clinical efficacy [37]. In addition, several newer systemic agents have been developed or approved that target specific Th2-associated cytokines, including IL-13 and IL-31, to reduce inflammation and alleviate symptoms such as pruritus and erythema [38,39]. Formulations with an acidic pH are also recommended to optimize skin permeability and enhance antimicrobial defense. Collectively, these therapeutic strategies aim to disrupt the cycle of inflammation and barrier impairment that perpetuates AD [40].

In recent years, there has been a growing interest in non-pharmacological approaches and complementary therapies for AD [36,41,42,43]. Among these approaches, hot springs have long been recognized for their therapeutic benefits in treating various skin conditions, including AD, due to their unique mineral content and thermal properties. This form of therapy has been used for centuries and remains a popular treatment for dermatologic conditions to date [44,45,46]. Research indicates that hot spring therapy can improve the condition of AD in approximately 75% of patients [47]. Additionally, the minerals in hot springs, such as sulfur, selenium, and magnesium, have anti-inflammatory and antioxidant properties that can reduce skin inflammation, itching, and redness associated with AD [44,47]. Immersion in hot spring water also enhances skin hydration and barrier function by increasing the absorption of moisture and nutrients, thereby helping to restore the natural protective barrier of the skin. The relaxing and stress-relieving effects of hot springs can further contribute to managing AD symptoms, as stress is known to exacerbate skin conditions [48,49].

A few studies have been conducted to understand the mechanisms by which hot spring treatment can influence AD pathology. Most of the studies suggest that the temperature and combination of minerals in the hot-spring water show some anti-inflammatory effects, that are indirectly beneficial for atopic conditions. For example, a study showed that hot spring treatment decreases the Th2 response, including IL-4 production, and increases the T-reg response in an AD murine model [49]. It has also been shown to decrease the skin bacterial population, which could be an important factor in AD pathology [44]. However, the minerals in hot spring water could play a direct role in skin health and AD pathology. For instance, selenium, a mineral rich in hot spring water, is shown to protect keratinocyte stem cells against senescence by preserving their stemness phenotype and maintaining skin health [50]. Therefore, we hypothesized that hot spring water might have a direct role in regulating AD pathology. Each of the hot spring water has an unique combination of mineral content. Hence, this study focuses on investigating the role of hot spring water from the Arifuku hot spring in Shimane prefecture, Japan, on barrier function and the underlying mechanisms using an AD mouse model.

## 2. Materials and Methods

### 2.1. Animals

Adult male hairless mice of 2 months of age were purchased from Jackson Laboratory (Yokohama, Kanagawa, Japan) and housed in a temperature and humidity-controlled animal facility under specific pathogen free (SPF) conditions with 12 h light-dark cycle. Two to three mice were housed in a plastic cage where wood dust was used as bedding. The mice received normal rodent chow and water ad libitum. After purchase, the mice were housed in the facility for at least 1 week for acclimatization.

### 2.2. Generation of AD Mouse Model

All animal experimental protocols were reviewed and approved by the Ethical Committee of the Shimane University School of Medicine (approval number: IZ5-35). Eight-week-old hairless mice were used to establish an injury-induced AD model. The mice were housed in a controlled environment with a 12-h light-dark cycle, constant temperature (22 ± 2 °C), and humidity. To induce skin barrier disruption and simulate AD, the tape-stripping method was employed. Briefly, the dorsal skin of each mouse was gently shaved, and the left side was subjected to repeated tape-stripping using adhesive tape (approximately 10–15 times) until mild erythema was observed. The right side of the shaved dorsal skin of the same animal was used to assess skin changes in the non-tape-stripped area. The integrity of the skin barrier in both tape-stripped and the non-tape-stripped areas was assessed by measuring transepithelial water loss (TEWL) before and after tape-stripping. Mice were then divided into two groups: one group received daily topical applications of hot spring water, while the other was treated with tap water. Skin samples were collected at designated time points for histological and immunological analysis. In this study, five mice were allocated to each treatment group at each time point.

### 2.3. Preparation and Treatment with Tap Water and Hot Spring Water

Hot spring water was collected from Arifuku hot spring in Shimane prefecture, Japan, and allowed to cool to room temperature (25 ± 2 °C). Tap water was also collected for comparison. Both water samples were sterile filtered using a 0.22 µm filter unit (Millipore, Burlington, MA, USA) before application. About 1 mL of filtered hot-spring water or tap water was then applied to the tape-stripped and non-tape-stripped areas of the skin of the mice using a liquid sprayer once daily for the designated duration (0, 1, 2 and 3 days).

### 2.4. Measurement of Transepithelial Water Loss (TEWL)

Dermal water content and TEWL were measured using the DermaLab USB module (Cortex Technology, Hadsund, Denmark), as previously described (Sivaprasad et al., 2010 [51]). Briefly, the measurements were performed in a controlled environment to minimize external influences. The TEWL probe was placed on the dorsal skin of each mouse, and readings were recorded after stabilization. An average of two readings was used for each mouse to ensure accuracy. All measurements were conducted by the same investigator to maintain consistency.

### 2.5. Preparation of Skin Samples for Staining

After completing the treatment, mice were deeply anesthetized using Isoflurane (Pfizer, New York, NY, USA) and transcardially perfused with normal saline, followed by 4% paraformaldehyde (PFA) in 0.1 M phosphate buffer (pH 7.4). Dorsal skin samples were collected from both the tape-stripped (left side) and healthy sides (right side). The samples were then post-fixed in 4% PFA overnight. Following fixation, the tissues were cryoprotected in a 30% sucrose solution in a 0.1 M phosphate buffer (pH 7.4). The cryoprotected tissues were embedded in an optimal cutting temperature (OCT) compound, and 6 µm thick sections were prepared for histological staining or immunostaining.

### 2.6. Histological Examination

Skin sections were stained with hematoxylin and eosin (H&E) to evaluate histological changes. Briefly, the 6 µm frozen sections were rinsed in distilled water and stained with hematoxylin solution (Gills hematoxylin No. 2, Wako, Osaka, Japan) for 5 min. After washing in running tap water for 20 min, the sections were counterstained with eosin for 2 min, dehydrated through graded ethanol solutions, cleared in xylene, and mounted with a coverslip using a synthetic mounting medium. Histological changes, including epidermal thickness and stratum spinosum alterations, were examined under a light microscope (Nikon, ECLIPSE, E600, Tokyo, Japan). The thickness of stratum spinosum was analyzed using NIS-Elements D 3.1 (Nikon).

### 2.7. Immunostaining

Immunostaining was performed to assess the expression of filaggrin, transient receptor potential vanilloid 4 (TRPV4), CD8, and IL-4 in skin sections. The 6 µm frozen sections were washed in phosphate-buffered saline (PBS, pH 7.4) for 5 min. For antigen retrieval, sections were incubated in citrate buffer (pH 6.0) at 95 °C for 20 min, then allowed to cool down for another 20 min. After cooling, sections were washed with PBS and blocked with 5% normal goat serum for 30 min at room temperature to reduce nonspecific binding. During filaggrin immunostaining, sections were incubated in 0.3% H_2_O_2_ to inhibit the endogenous peroxidase activity, the overnight at 4 °C with a primary anti-filaggrin antibody (rabbit, 1:100, Abcam, Cambridge, MA, USA), followed by incubation with a biotin conjugated anti-rabbit IgG for 1 h at room temperature. Then, the tissue section was treated with an ABC complex (Vector, Newark, CA, USA), signal was developed using the 3,3-diaminobenzidine (DAB, Sigma, St. Louis, MO, USA) method, and examined under a light microscope. For TRPV4 and CD8 detection, sections were incubated overnight at 4 °C with primary antibodies against TRPV4 (rabbit, 1:100, Abcam) and CD8 (mouse, 1:50, SantaCruz, Dallas, TX, USA), followed by incubation with a Texas Red-conjugated secondary antibody for 1 h at room temperature. For IL-4 detection, sections were incubated with an anti-IL-4 primary antibody (mouse, 1:100, SantaCruz) and subsequently with an FITC-conjugated secondary antibody. After staining, the tissue sections were examined under a fluorescence microscope (Nikon). The immunopositive areas were quantified using ImageJ software (version ij154-win-java8).

### 2.8. Statistical Analysis

All numerical data are presented as mean ± SD. Comparisons between groups were analyzed using one-way ANOVA followed by Scheffé’s post hoc test or an independent *t*-test, as appropriate. A *p*-value of less than 0.05 was considered statistically significant. All statistical analyses were performed using SPSS (version 27) software.

## 3. Results

### 3.1. Properties and Mineral Content of the Hot-Spring Water Used Are Described Below

First, the properties of hot-spring water were analyzed. The hot-spring water was colorless, astringent in taste, and the temperature was 45.3 °C. The pH is slightly acidic at 6.3, and the density was 1.0041 g/cm^3^. Analysis of cationic minerals revealed that Na^+^ was high (1710 mg/kg), followed by Ca^2+^ (433 mg/kg), Mg^2+^ (84.4 mg/kg), and K^+^ (72.5 mg/kg) (Table 1). Also, a considerable amount of Sr^2+^ (12.3 mg/kg) was contained in the hot-spring water (Table 1). Among the anions, Cl^−^ was highest in amount (2660 mg/kg), followed by SO4^−^ (965 mg/kg) and HCO3^−^ (941 mg/kg) groups (Table 2). Among the non-dissociated compounds, the amount of H_2_SiO_3_ was highest (118 mg/kg), followed by HBO_2_ (35.3 mg/kg) and HAsO_2_ (2.3 mg/kg) (Table 3). As dissolved gas, CO_2_ was detected in the hot-spring water (Table 4). The levels of other trace elements are shown in Table 5.

### 3.2. Effects of Hot-Spring Water on the Skin Barrier Function in an Injury-Induced AD Model Mouse Are Described Below

**Figure 1:**
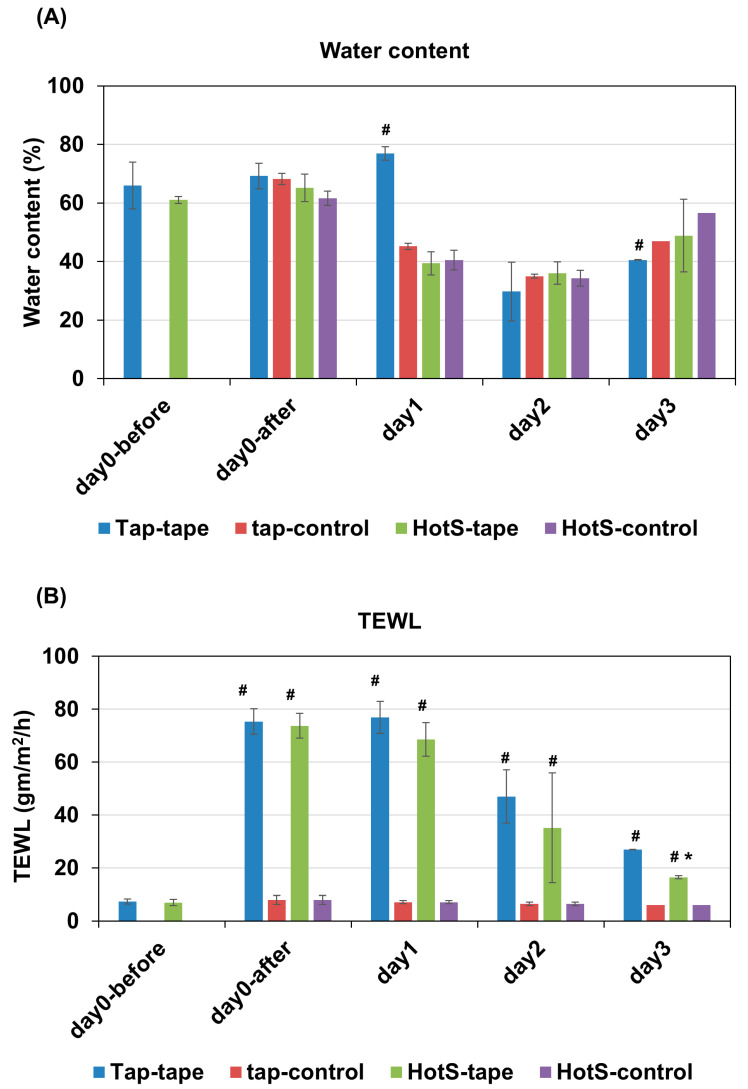
Effects of hot-spring water on the water content and trans-epidermal water loss in an AD mouse model. AD model was generated by tape-stripping, and treated with tap water, or hot-spring water for indicated times. Water content (**A**) and trans-epidermal water loss (TEWL) (**B**) was measured in tape-striped and non-tape-stripped control skin daily for 3 days, as described in the Materials and Methods. Data is presented here as average ± SD (n = 5). Tap-tape = tape-stripped area of the mice treated with tap water; Tap-control= non-tape-stripped area of the mice treated with tap water; HotS-tape = Tape-stripped area of the mice treated with hot-spring water; HotS-control = Non-tape-stripped area of the mice treated with hot-spring water. ^#^ *p* < 0.01 vs. corresponding control; * *p* < 0.01 vs. corresponding Tap-tape.

To understand the impact of tap water (tap water group) and hot-spring water (hot-spring water group) on skin barrier functions in AD model mice, we measured the skin water content and trans-epidermal water loss (TEWL) in a time-dependent manner. Initially, the results indicated that the water content remained relatively stable immediately after tape-stripping injury (day 0) in both mouse groups (Figure 1A). However, on day 1, the water content had significantly decreased in the tape-stripping injury, and in non-tape stripped areas in all groups, except in the tape stripped areas of the mice treated with tap water, where it remained elevated. On day 2, the water content had significantly decreased in both the tape-stripped and non-tape-stripped areas across all groups, compared to day 0 (Figure 1A). This trend continued into day 3, when the water content was still lower than that of day 0 in all groups, both in the tape-stripped and non-tape-stripped areas (Figure 1A). Notably, the tape-stripped areas of the tap-water-treated group exhibited a lower water content compared to their respective non-tape-stripped areas at day 3 (Figure 1A).

We also examined the rate of trans-epithelial water loss in these mice. The results indicated a significant increase in TEWL in the tape-stripped areas compared to the non-tape stripped areas of both tap-water-treated and hot-spring-water-treated mice on days 0 and 1 (Figure 1B). From day 2 onward, TEWL began to decrease in tape-stripped areas in both the tap-water-treated and hot-spring-water-treated groups, with this trend persisting into day 3 (Figure 1B). Crucially, by day 3, the TEWL from the tape-stripped areas in the hot-spring-water-treated group was significantly decreased compared to that in the tap-water-treated group (Figure 1B).

### 3.3. Effects of Hot-Spring Water on the Skin Histology in an AD Model Mouse

**Figure 2:**
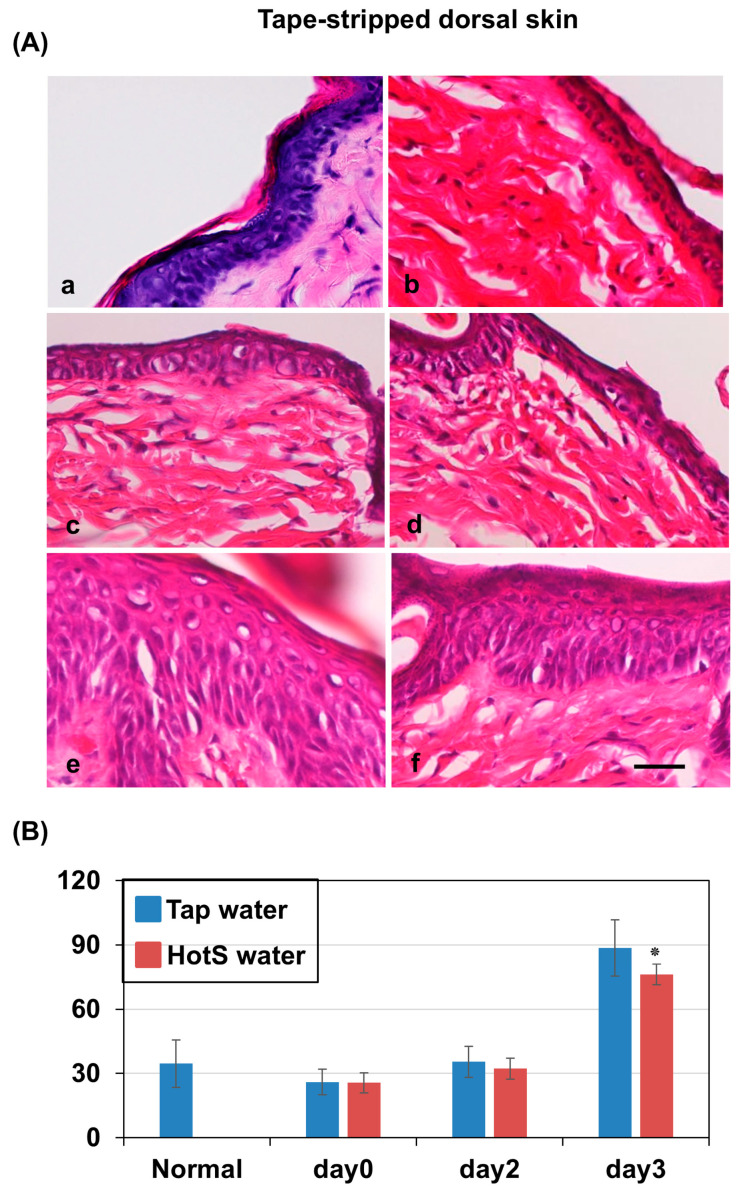
Time-dependent histological evaluation of the skin on the tape-stripped areas of AD mouse model treated with tap water or hot-spring water. AD mouse models were generated by tape-stripping, and histological evaluation of the tape-stripped areas were done by Haematoxylin and Eosin (HE) staining, as described in the Materials and Methods. (**A**) Representative HE photomicrographs of the mice skin at day 0 (**b**), day 2 (**c**,**d**), and day 3 (**e**,**f**) after tape-stripping are shown here, where (**c**,**e**) are from the mice that received tape water treatment, and (**d**,**f**) received hot-spring water. All pictures were taken at ×400 magnification. Figure (**a**) is a representative photomicrograph of the skin of a normal mouse. (**B**) The thickness of stratum spinosum was measured, and the averages thicknesses are shown here. Statistical significance is denoted as follows: * *p* < 0.05 vs. corresponding tap-water-treated mice. HotS = hot-spring water treated mice. Scale bar = 50 μm.

Next, we analyzed the time-dependent changes in the skin histology of AD model mice after treatment with tap water or hot-spring water. The hematoxylin and eosin staining results showed that the normal skin of the mice displayed distinct layers in the epidermis: stratum basale, stratum spinosum, stratum granulosum, and stratum corneum (Figure 2A(a)). After tape-stripping, there were minimal changes in the epidermis, except the detachment or removal of stratum conium (Figure 2A(b)). In the tape-stripped areas of skin treated with tap water and hot-spring water, there were minimal changes in the stratum basale at day 0 (Figure 2A(b)). Similarly, the stratum spinosum and stratum granulosum showed little change on day 0. From day 2, the stratum spinosum layer in tape-stripped areas began to expand, with some vacuolation appearing in both the tap-water-treated and hot-spring-water-treated mice (Figure 2A(c,d)). By day 3, the stratum spinosum was significantly thicker in the tap-water-treated mice compared to those treated with hot-spring water (Figure 2A(e,f),B). Additionally, numerous vacuolations were observed in this layer in the tap-water-treated mice, while very few were seen in the hot-spring-water-treated mice. Importantly, the stratum granulosum was well-formed in the tape-stripped areas of hot-spring-water-treated mice, whereas it was very thin in the tap-water-treated mice (Figure 2A(e,f)). The stratum corneum was found to be detached or absent on days 0 and 2. By day 3, the stratum corneum began to form in the tape-stripped areas of the hot-spring-water-treated mice, a feature that was difficult to observe in the tap-water-treated mice (Figure 2A(e,f)). On day 7, the thickness of stratum spinosum at the tape stripped areas of tap-water-treated mice was decreased similar to hot-spring-water-treated mice.

**Figure 3:**
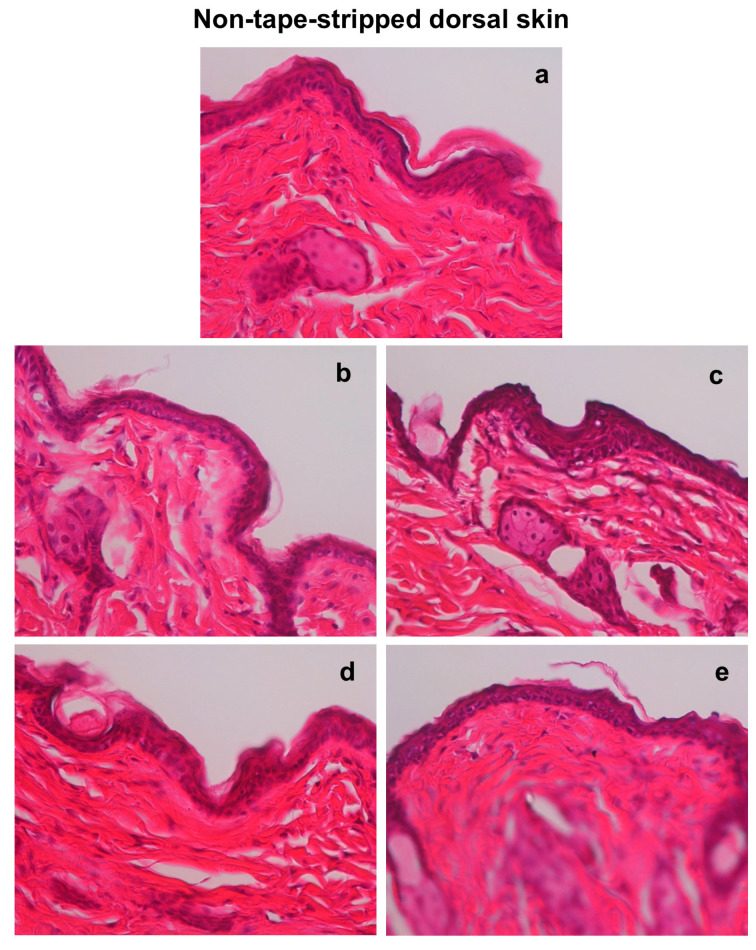
Time-dependent histological evaluation of the skin on the non-tape-stripped areas of AD mouse model treated with tap water or hot-spring water. AD models were generated by tape-stripping, and histological evaluation of the non-tape-stripped control areas were done by Haematoxylin and Eosin (HE) staining, as described in the Materials and Methods. Representative HE photomicrographs of the mice skin of non-tape-stripped control areas at day 0 (**a**), day 2 (**b**,**c**), and day 3 (**d**,**e**) after tape-stripping are shown here, where (**b**,**d**) are from the mice that received tape water treatment, and (**c**,**e**) received hot-spring water. All pictures were taken at ×400 magnification. Scale bar = 50 μm.

Next, we examined the time-dependent changes in the skin histology of non-tape-stripped areas in mice treated with tap water and hot spring water. Although there were almost no changes observed on day 0 (Figure 3a), the thickness of the stratum spinosum layer began to increase by day 2 in the hot-spring-water-treated mice only (see Figure 3b,c). In contrast, the stratum spinosum layer in the tap-water-treated mice increased in thickness by day 3 (Figure 3d), whereas it returned to normal thickness in the hot-spring-water-treated mice on day 3 (Figure 3e). However, there were no statistically significant differences in stratum spinosum thickness between tap-water- and hot-spring-water-treated mice in the non–tape-stripped areas at any time point.

### 3.4. Effects of Hot-Spring Water on TRPV4 Protein Levels in an AD Model Mouse

**Figure 4:**
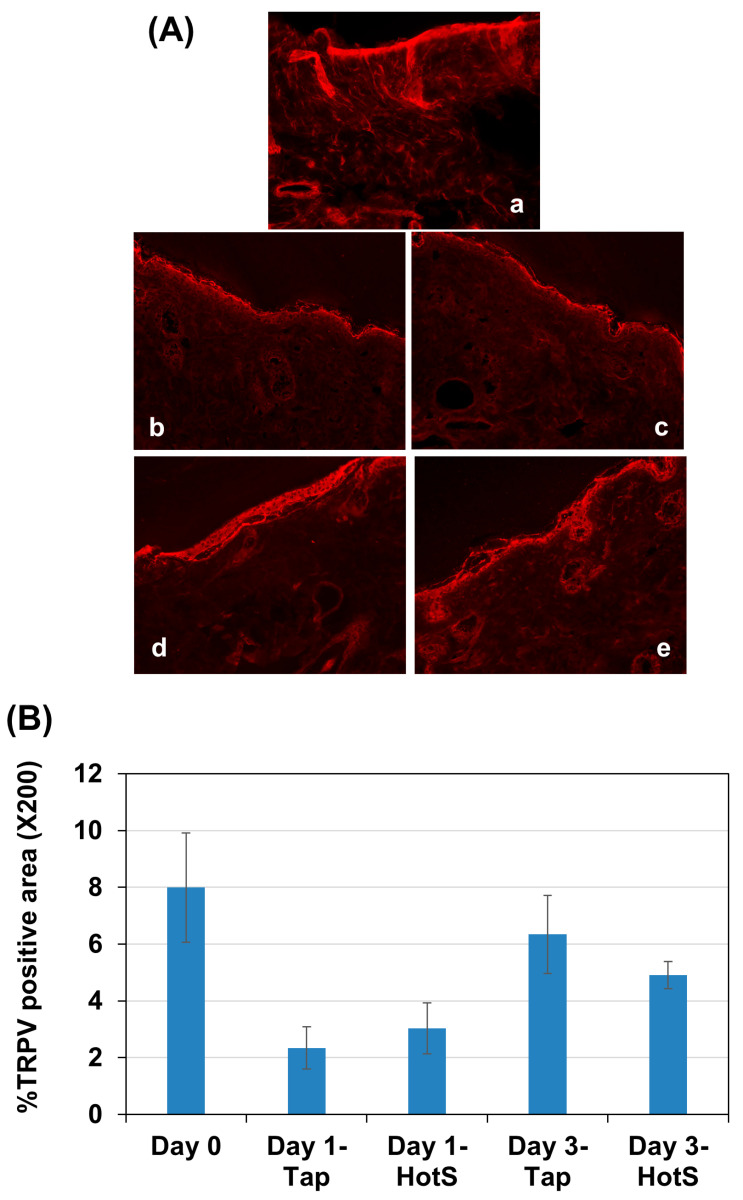
Time-dependent evaluation of TRPV4 protein levels in the skin of an AD mouse model treated with tap water or hot-spring water. AD models were generated by tape-stripping, and TRPV4 protein levels in the skin of tape-stripped areas were evaluated by immunostaining, as described in the Materials and Methods. (**A**) Representative TRPV4 immunostaining photomicrographs of the mice skin of tape-stripped areas at day 0 (**a**), day 1 (**b**,**c**), and day 3 (**d**,**e**) are shown here, where (**b**,**d**) are from the mice that received tap water treatment, and (**c**,**e**) received hot-spring water. All pictures were taken at ×400 magnification. Scale bar = 50 μm. The immuno-positive areas were measured using ImageJ, expressed as % of total area, and the average data were presented in (**B**).

Since TRPV4 is known to be involved in the pathology of AD, we examined the time-dependent changes in its levels in AD model mice. Immunostaining results indicated that TRPV4 is primarily expressed in the epidermal layer of the skin. On day 1, the levels of TRPV4 decreased in the tape-stripped areas of both tap-water-treated and hot-spring-water-treated mice compared to day 0 (Figure 4A(a–c)). By day 3, the levels of TRPV4 almost had returned to those observed on day 0 in the tape-stripped areas of both tap-water-treated and hot-spring-water-treated mice (Figure 4A(d,e)). Quantification of the immunostaining also showed no difference in the immunopositive areas between the tap-water-treated group and the hot-spring-water-treated group at the same time point (Figure 4B).

### 3.5. Effects of Hot-Spring Water on Filaggrin Protein Levels in an AD Model Mouse

**Figure 5:**
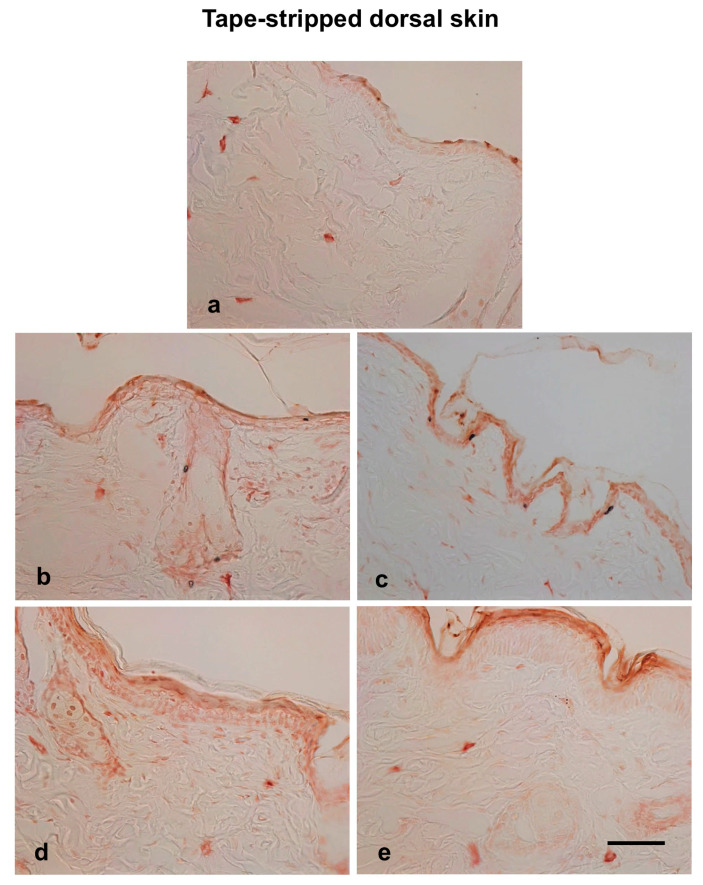
Time-dependent evaluation of filaggrin protein levels in the tape-stripped skin areas of an AD mouse model treated with tap water or hot-spring water. AD models were generated by tape-stripping, and filaggrin protein levels in the skin of tape-stripped areas were evaluated by immunostaining, as described in the Materials and Methods. Representative filaggrin immunostaining photomicrographs of the mice skin of tape-stripped areas at day 0 (**a**), day 2 (**b**,**c**), and day 3 (**d**,**e**) are shown here, where (**b**,**d**) are from the mice that received tap water treatment, and (**c**,**e**) received hot-spring water. All pictures were taken at ×400 magnification. Scale bar = 50 μm.

**Figure 6:**
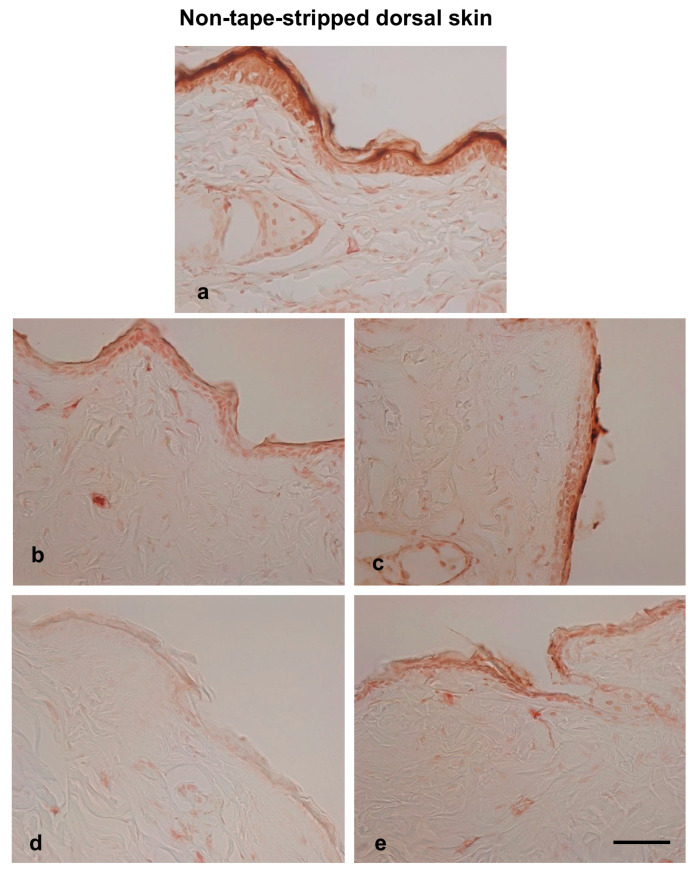
Time-dependent evaluation of filaggrin protein levels in the non-tape-stripped control skin areas of an AD mouse model treated with tap water or hot-spring water. AD models were generated by tape-stripping, and filaggrin protein levels in the skin of non-tape-stripped control areas were evaluated by immunostaining, as described in the Materials and Methods. Representative filaggrin immunostaining photomicrographs of the mice skin of tape-stripped areas at day 0 (**a**), day 2 (**b**,**c**), and day 3 (**d**,**e**) are shown here, where (**b**,**d**) are from the mice that received tap water treatment, and (**c**,**e**) received hot-spring water. All pictures were taken at ×400 magnification. Scale bar = 50 μm.

Filaggrin plays a crucial role in skin barrier function. Given that both trans-epidermal water loss (TEWL) and histological examinations showed an improvement in skin barrier function following hot spring water treatment, we investigated the levels of filaggrin protein in the skin of injury-induced AD model mice. Immunostaining results revealed that filaggrin was primarily expressed in the stratum corneum and stratum granulosum, although some cells in deeper layers also showed its expression (Figure 5 and Figure 6a). Following tape stripping, the number of filaggrin-expressing cells in the stratum granulosum decreased compared to non-tape stripped areas at day 0 (Figure 5a). The levels of filaggrin increased on day 2 compared to day 0 in the tape-stripped areas for both tap-water-treated and hot-spring-water-treated mice (Figure 5b,c). By day 3, filaggrin levels in the tape-stripped areas had increased further compared to days 0 in both groups of mice (Figure 5d,e). In the hot-spring-water-treated mice, the protein immunoreactivity was predominantly strong in the upper layers, including the stratum granulosum (Figure 5e). In contrast, in the tap-water-treated mice, the immunoreactivity was more diffuse, with less pronounced positivity in the upper layers of the epidermis (compare 5d and 5e).

On the non-tape-stripped side, filaggrin levels were highest on day 0, with strong immunopositive areas found in the stratum granulosum and stratum corneum (Figure 6a). Interestingly, in tap water-treated mice, filaggrin levels gradually decreased on days 2 and 3 in this area (Figure 6b,d). In the case of hot-spring water-treated mice, filaggrin levels also decreased over time (Figure 6c,e). However, on day 3, filaggrin remained strongly positive in the stratum granulosum and stratum corneum of hot-spring-water-treated mice, which was not observed in tap-water-treated mice (compare Figure 5d,e). Moreover, on day 3, the levels of filaggrin in the non-tape-stripped areas were higher in hot-spring-water-treated mice compared to tap-water-treated mice (Figure 6d,e).

### 3.6. Effects of Hot Spring Water on Skin Inflammation in an AD Mouse Model

**Figure 7:**
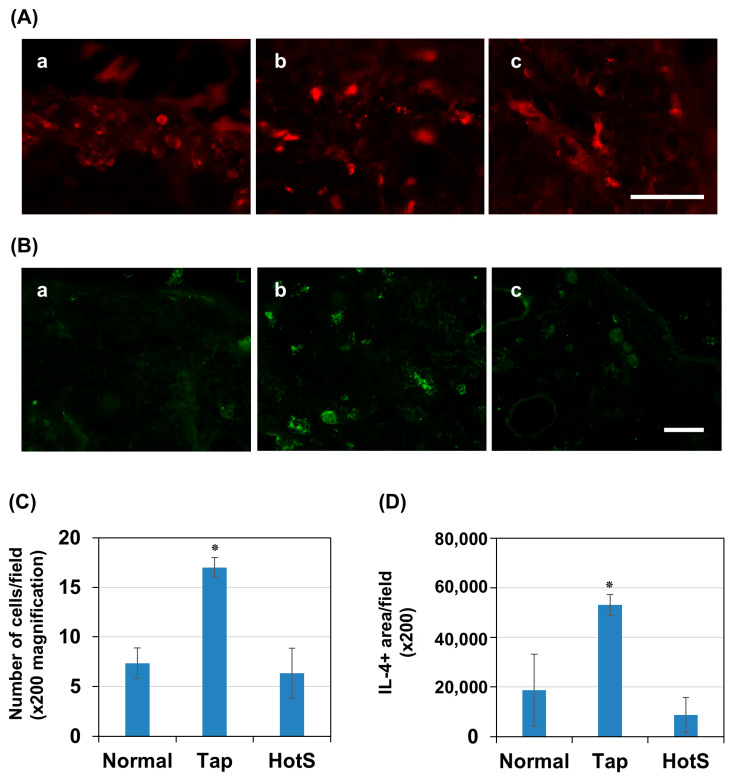
Time-dependent evaluation of CD8+ T cells and IL-4 levels the skin of an AD mouse model treated with tap water or hot-spring water. AD models were generated by tape-stripping, and CD8+ cell number and IL-4 immunopositive areas in the skin of tape-stripped areas were evaluated by immunostaining, as described in the Materials and Methods. (**A**) Representative CD8 immunostaining photomicrographs of normal mice skin (control) (**a**), tape-stripped and tap-water treated (**b**), and tape-stripped and hot-spring water treated (**c**) are shown here. All pictures were taken at ×200 magnification. Scale bar = 100 μm. CD8+ cells were counted using ImageJ, expressed as number of cells per field, and the average data were presented in (**C**). (**B**) Representative IL-4 immunostaining photomicrographs of normal mice skins (normal) (**a**), tape-stripped and tap-water treated (**b**), and tape-stripped and hot-spring water treated (**c**) skins at day 3 are shown here. All pictures were taken at ×200 magnification. Scale bar = 100 μm. IL-4 positive areas were quantified using ImageJ, expressed as positive areas per field (pixel), and the average data were presented in (**D**). Statistical significance is denoted as follows; * *p* < 0.01 vs. normal or hot-spring-water-treated (HotS) mice.

Since filaggrin levels in the skin are greatly influenced by inflammation, we investigated the effects of hot spring water on CD8^+^ T cells and IL-4 cytokines in the tape-stripped areas three days after model induction. Immunostaining results showed that CD8^+^ T cells were rarely detected in normal skin, but were significantly increased in number three days after tape stripping in mice treated with tap water (Figure 7A,C). Notably, treatment with hot spring water significantly reduced the number of CD8^+^ cells to a level comparable to that of normal skin

Next, we examined IL-4 levels, a cytokine known to increase in AD and contribute to reduced filaggrin expression. Similarly, immunostaining results demonstrated that IL-4-positive areas were rarely detected in normal skin but were significantly increased three days after tape stripping in tap-water-treated mice (Figure 7B,D). Importantly, treatment with hot spring water reduced IL-4-positive areas to levels comparable to those observed in normal skin.

## 4. Discussion

The most significant findings of this study are that filaggrin expression increased in the upper layer of the epidermis on the tape-stripped side of injury-induced AD model mice when treated with hot spring water, which is accompanied by a well-formed stratum granulosum. Skin barrier function is crucial in the pathology of AD, serving as the first line of defense against environmental irritants, allergens, and pathogens [52]. In individuals with AD, this barrier is typically compromised, leading to increased permeability and reduced protection, particularly against allergens and irritants, which induce an inflammatory condition [53]. Furthermore, a weakened skin barrier loses moisture more readily, contributing to the dry and scaly skin characteristic of the condition [52,53,54]. Given that the maturation of the stratum granulosum to stratum corneum and the role of filaggrin in aggregating keratin fibers are essential for skin barrier formation, our data suggests that Arifuku hot spring water can improve skin barrier function. Thus, maintaining and restoring the integrity of the skin barrier through hot spring treatment could be a key point in managing AD.

An assessment of tap water in Japan shows that its mineral content is generally low, resulting in soft water with a pH range of 5.8–8.6 [55]. In contrast, chemical analysis revealed a high concentration of sodium, calcium, magnesium, bicarbonate, chloride, phosphate, and sulfate ions in Arifuku hot spring water, especially with a notable amount of strontium. Therefore, the beneficial effects of hot-spring water on injury-induced AD may be attributed to its higher mineral content compared with tap water. Studies have shown that strontium can suppress sensory irritation without causing local anesthetic side effects, making it a potential treatment for irritant dermatitis [56]. In addition, strontium exhibits antioxidative and anti-inflammatory properties, and promotes keratinocyte growth and differentiation [57,58,59]. As a mechanism, strontium has been shown to modulate T-cell activity by regulating calcium signaling, thereby influencing signaling pathways such as NF-κB and the production of cytokines including TNF-α and IL-10. Through calcium signaling regulation, strontium further modulates the differentiation of murine keratinocytes and increases the expression of filaggrin [60]. Accordingly, strontium salts have been demonstrated to improve skin barrier function in skin irritation injury models in both animals and humans [57,61,62]. While we did not specifically investigate whether the presence of strontium alone is responsible for these beneficial effects, other chemicals in this hot-spring water, such as silicic acid, have also been shown to benefit the skin by improving thickness and reducing wrinkles [63]. Also, a high concentration of bicarbonate could be important for the commensal skin bacterial population. Therefore, it is possible that the beneficial effects observed may stem from a combination of chemicals present in the hot spring water, rather than solely from strontium [64]. However, it will be interesting to investigate which chemicals of the hot spring water provide beneficial effects. Furthermore, it is worth noting that the beneficial effects are not solely attributed to the temperature of the hot-spring water, as we applied the water at room temperature during our study.

The measurement of skin water content showed that on day 1, the water content of the skin in tape-stripped areas of mice treated with tap water increased significantly compared to all other conditions. However, by day 2, the water content had gradually decreased in all conditions, only to begin increasing again by day 3. The initial decrease in water content can likely be attributed to diminished barrier functions caused by the loss of stratum granulosum due to tape-stripping. However, the explanation for the increased water content on day 1 in the tape-stripped areas of tap-water-treated mice is difficult to ascertain. Research on the water content of skin with dermatitis reveals some interesting yet conflicting findings. Some studies indicate an increase in water content in the upper layers of individuals with dermatitis [65,66,67]. However, this water appears to be trapped within the inflamed tissue rather than being properly integrated into healthy skin cells [66,67,68]. This trapped water disrupts the natural barrier function of the skin, which normally helps retain moisture and keep irritants out [69]. Therefore, it seems that hot-spring water may have an anti-inflammatory effect on atopic skin conditions from the outset, reducing inflammation and resulting in reduced water content in the skin from the beginning.

Another inconsistency is observed in the non-tape-stripped areas of both hot spring water and tap-water-treated mice, where the water content decreases similarly to the tape-stripped areas. We found that in these non-tape-stripped areas, there is reactive thickening of the stratum spinosum, along with decreased filaggrin expression. These results suggest that there is a reaction to the tape-stripping in other parts of the skin as well, which may be responsible for the decreased water content. However, Trans-Epidermal Water Loss (TEWL) results did not indicate increased water loss from the non-tape-stripped areas, suggesting that despite decreased filaggrin expression, the barrier remains intact in those areas [70,71]. TEWL results also revealed that water loss gradually decreased in the tape-stripped areas from day 2 in both tap-water-treated and hot-spring-water-treated mice. Importantly, hot spring water significantly decreased water loss compared to tap-water-treated mice in the tape-stripped areas by day 3, suggesting that hot-spring water has a healing effect on skin inflammatory conditions.

Histological examination revealed that tape-stripping completely removed, or at least detached, the stratum corneum. Time-dependent histological analysis of the skin showed typical changes consistent with AD [72]. We found that after tape stripping, the stratum spinosum increased in thickness in both tap-water-treated and hot-spring-water-treated areas. However, on day 3, the thickness of the stratum spinosum decreased in hot-spring-water-treated mice compared to tap-water-treated mice. The increased thickness is usually caused by inflammatory reactions involving growth factors and cytokines, including IL-4 and IL-13 [25,73,74,75]. Also, inflammatory conditions prevent the keratinocytes from maturing and forming the upper barrier layer [26,57]. Although we did not investigate the inflammatory component in AD and the effects of hot spring water on it, it is possible that hot spring water might decrease inflammation and provide beneficial effects. As evidence of decreased inflammation, we found that vacuolation, which is typically present in AD inflammatory conditions, was reduced. However, minerals like strontium are known to increase keratinocyte proliferation and maturation [57]. Since the concentration of strontium is high in the hot spring water, it might help keratinocytes to mature, leading to the decreased thickness of the stratum spinosum. Indeed, we found that the stratum granulosum was well-formed in the hot-spring-water-treated tape-stripped area only. Such a well-formed upper layer helps to regain the barrier function of the skin, an important aspect of AD therapy.

Another important finding is that hot spring water increases filaggrin expression in tape-stripped areas. The expression of filaggrin, a key protein in skin barrier function, is tightly regulated at the molecular level, particularly in keratinocytes [10]. Several signaling pathways modulate its expression. One of the prominent regulators is calcium signaling, where elevated intracellular calcium levels trigger filaggrin expression through the activation of calcium-dependent transcription factors like CREB [63,64,76,77]. Additionally, cytokines such as IL-4 and IL-13, prevalent in AD, can downregulate filaggrin expression via the JAK-STAT pathway [24,78]. Our investigation into inflammation status also suggests such a possibility. The significant increase in CD8^+^ T cells and IL-4 expression following tape stripping in tap-water-treated mice indicates that immune activation and Th2 cytokines play a crucial role in AD pathology [28,29]. The reduction of CD8^+^ T cells and IL-4 levels in hot-spring-water-treated mice suggests that this treatment may help suppress excessive immune activation and mitigate inflammatory responses in the skin. Given that IL-4 is known to downregulate filaggrin expression [8,25,79], the observed decrease in IL-4-positive areas in hot-spring-water-treated mice may contribute to improved skin barrier function. Hot spring water contains a high concentration of strontium, which has been shown to increase filaggrin expression in cell culture systems, likely through the regulation of calcium signaling [57]. Hot spring water also contains a high concentration of bicarbonate. Taking a bath in bicarbonate water has been shown to increase blood flow by enhancing eNOS activity and NO levels [80]. This increased blood flow might positively affect the inflammatory condition and improve the skin’s condition in AD. Additionally, it will be interesting to see the effects of strontium on skin inflammation, as this mineral is present in high levels in hot spring water.

TRPV4, a member of the transient receptor potential (TRP) family of ion channels, plays a multifaceted role in AD (AD) [9]. It is expressed in various skin cell types, including keratinocytes, macrophages, and sensory neurons, where it regulates inflammatory signaling, barrier integrity, and itch sensations [9,14]. In macrophages, TRPV4 has been shown to inhibit NF-κB, thereby reducing the expression of inflammatory cytokines. TRPV4 is also a crucial component of skin keratinocytes, where it contributes to calcium influx and downstream signaling cascades that regulate epidermal differentiation and skin barrier homeostasis [9,14,81]. Research has shown that TRPV4 expression is upregulated in the skin of individuals with AD, and this upregulation is associated with increased inflammatory responses and skin barrier dysfunction [9]. Its activation leads to calcium influx, triggering downstream signaling cascades implicated in inflammation, barrier dysfunction, and itch sensation, all hallmark features of AD [81]. As a consequence of the activation of inflammatory signaling, the release of proinflammatory cytokines such as IL-6 and IL-8 from keratinocytes and immune cells are increased [9,82]. Furthermore, TRPV4 activation has been linked to the disruption of epidermal barrier function, contributing to increased permeability and susceptibility to allergens and irritants in AD-affected skin [7]. However, in the present study, TRPV4 expression levels were not significantly altered by hot-spring water treatment, suggesting that the beneficial effects of hot-spring water in this AD model are likely independent of TRPV4-mediated pathways. Instead, other mechanisms, such as modulation of inflammatory cytokines or enhancement of skin barrier proteins, may account for the observed improvements.

There are several limitations to this study. First, we used a skin injury model through tape stripping without applying any allergen to the injured skin. Since no allergen was used to elicit an immune response, such a model might not be considered as an AD model. However, we used hairless mice, which exhibit splenomegaly and waxy skin deposits that peel off in large flakes, suggesting an altered immune system that affects the skin [83]. Tape-stripping injuries on these mice have caused desquamation and the formation of shallow furrows on the skin, appearing as fine, regular wrinkles, which are indicative of chronic eczematous dermatitis [84]. Also, tape stripping injury even in C57/BL mice can elevate several Th2-type cytokines [84,85]. Since Th2 type immune response plays an important role in AD, it is conceivable that tape stripping in the hairless mice model may share some characteristics with AD. However, further detailed studies are necessary to understand the underlying pathophysiology of tape stripping in the context of AD pathology in hairless mice. Second, we have shown that the levels of the skin barrier protein filaggrin were increased by our hot spring water. However, the detailed molecular mechanisms behind these beneficial effects have not been studied. We examined changes in Th2-type cytokines and CD8^+^ T cells, which are known to contribute to decreased filaggrin levels in the skin, particularly in conditions like AD. However, the direct effects of our hot spring water on CD8^+^ T cell accumulation and Th2-type cytokine production require further in vitro and in vivo studies. Third, we have only examined the initial changes in skin pathology after tape stripping. Our preliminary experiments showed that, by seven days after model induction, the skin injury had significantly improved even in tap-water-treated mice. Therefore, a more comprehensive study monitoring time-dependent changes in skin pathology over a longer period, and across various AD models, would be valuable. Given that multiple AD models explore different aspects of disease pathology, it would be particularly interesting to evaluate the effects of our hot spring water in these other models of AD.

## 5. Conclusions

In conclusion, we have demonstrated that hot spring water can improve AD pathology, enhance the maturation of the stratum granulosum, and increase barrier protein filaggrin levels. Since barrier function disturbance is considered one of the main causes of AD, therapy with hot-spring water could provide an alternative therapeutic option for patients with AD.

## Figures and Tables

**Table 1:** Minerals content in the hot-spring water (Cations).

Components	mg	mval	mval%
Li^+^	1.8	0.26	0.25
Na^+^	1710	74.38	70.51
K^+^	72.5	1.85	1.75
Mg^2+^	84.4	6.94	6.58
Ca^2+^	433	21.61	20.49
Sr^2+^	12.3	0.28	0.27
Mn^2+^	0.4	0.01	0.01
Fe^2+^+Fe^3+^	4.1	0.15	0.14
Total Cations	2318.5	105.48	100.00

Table footnote: The amount of each mineral is contained in 1 kg of hot-spring water.

**Table 2:** Mineral content in the hot-spring water (Anions).

Components	mg	mval	mval%
F^−^	1.0	0.05	0.05
Cl^−^	2660	75.03	67.77
Br^−^	8.6	0.11	0.10
I^−^	0.7	0.01	0.01
SO_4_^2−^	965	20.09	18.14
HCO^3−^	941	15.42	13.93
Total Anions	4576.3	110.71	100.00

Table footnote: The amount of each mineral is contained in 1 kg of hot-spring water.

**Table 3:** Non-dissociated components in the hot-spring water.

Components	mg	mmol
HAsO_2_	2.3	0.02
H_2_SiO_3_	118	1.51
HBO_2_	35.3	0.81
Total non-dissociated components	155.6	2.34

Table footnote: The amount of each mineral is contained in 1 kg of hot-spring water.

**Table 4:** Dissolved gas components in the hot-spring water.

Components	mg	mmol
CO_2_	484	11.00
H_2_S	0.0	0.00
Total dissolved gas components	484.0	11.00

Table footnote: The amount of each mineral is contained in 1 kg of hot-spring water.

**Table 5:** Other trace elements in the hot-spring water.

Ba^2+^	0.05 mg
Al^3+^	0.03 mg
Cu^2+^	Not detected < 0.005 mg
Zn^2+^	0.088 mg
Cd^2+^	0.002 mg
Pb^2+^	0.073 mg
Hg	Not detected < 0.0005 mg
As	1.59 mg
S_2_O_3_^2−^	Not detected < 0.01 mg
CO_3_^2−^	Not detected < 0.1 mg

Table footnote: The amount of each mineral is contained in 1 kg of hot-spring water.

## Data Availability

All data of this study are used to prepare this manuscript.
